# COVID-19 quarantine: Psychological impact and support for children and parents

**DOI:** 10.1186/s13052-021-01005-8

**Published:** 2021-03-09

**Authors:** Francesco Demaria, Stefano Vicari

**Affiliations:** 1 Child and Adolescent Neuropsychiatry Unit, Bambino Gesù Children’s Hospital, IRCCS, Viale Ferdinando Baldelli, 41, 00146 Rome, Italy; 2 Department of Life Sciences and Public Health, Catholic University, 00168 Rome, Italy

**Keywords:** COVID-19, Children, Adolescents, Psychological impact

## Abstract

In response to the coronavirus (COVID-19) pandemic, national governments have imposed urgent sanitary and social measures to control the spread of the virus. One such measure is quarantine, which involves restricting people’s movement through the isolation of infected or suspected infected individuals in order to reduce the risk of new infections. Research has shown that quarantine is a psychologically stressful experience. With respect to children, lack of school and interruptions to daily routines could have a negative impact on their physical and mental health. Parents may also pass their psychological distress to children and practice inappropriate parenting behaviors, which could contribute to the development of post-traumatic stress symptoms in children.

In order to prevent these negative outcomes, governments must carefully consider any their decision to impose quarantine and family social care services must work together with children’s mental health services to ensure that the experience is as tolerable and safe as possible.

## Background

Uncertain and potentially dangerous situations such as epidemics (e.g. SARS, H1N1 and Ebola in recent history and the current COVID-19 pandemic) or traumatic experiences in the collective (e.g. hurricanes, earthquakes and acts of terror) elicit natural reactions such as confusion, fear and concern.

The present coronavirus (COVID-19) epidemic emerged in Wuhan, China, in December 2019; from March 2020, it spread steadily across the world [1]; on 9 March 2020, the Italian government announced a nationwide lockdown to contain the spread of the virus (www.governo.it/it/faq-iorestoacasa); and on 11 March 2020, the World Health Organization declared COVID-19 a pandemic (www.euro.who.int/.../2020/3/who-announces-covid-19-outbreak-a-pandemic).

As of 31 January 2021, the number of people who had been infected with COVID-19 (https://www.who.int/emergencies/diseases/novel-coronarovirus-2019) reached 102,083,344. Of these, 2,209,195 had been confirmed dead across 223 countries and territories.

## Main text

Since March 2020, urgent sanitary and social measures have been taken to contain the spread of the virus. One such measure is quarantine, which involves restricting people’s movement and is implemented through the isolation of Covid-19 positive individuals or individuals who have been in contact (or possible contact) with somebody who resulted to be positive in order to reduce the risk of new infections and to prevent viral transmission [2, 3]. Quarantine represents a sudden interruption to normal daily life. It breaks relationships and habits, resulting in disorientation and confusion. Throughout history, quarantines have been initiated in response to epidemics and disasters [4]. What have we learned about the consequences of this extraordinary measure, in relation to the psychological health of individuals?

A study published in 2003 regarding the SARS (severe acute respiratory syndrome) outbreak in Canada recorded a prevalence of post-traumatic stress symptoms in individuals following a short period of quarantine [5]. Furthermore, in 2014, quarantine measures in connection with the Ebola outbreak in rural Sierra Leone were found to result in psychological distress linked to cultural habits [6]. Finally, a Chinese study related to the current COVID-19 outbreak found that quarantine measures triggered a wide variety of psychological problems, such as panic disorder, anxiety and depression [7]. Thus, epidemics – and measures taken to contain them – may comprise psychologically stressful experiences.

If quarantine is a stressful experience for adults, we can imagine how difficult it may also be for children. Under quarantine, children must adapt their daily routines to an uncertain situation characterized by new and restricted rules, such as not attending school and not seeing their classmates. They must also confront new fears, potentially with a lot of unanswered questions.

We should not forget the healthcare workers children who have direct contact with the disease and who constitute the population most exposed and affected by Covid-19.

The special needs of children during epidemics and disasters are often unrecognized: What kind of experience might quarantine represent for children? How might the family intervene to reduce negative consequences for children during this uncertain and stressful period?

There are reasons to be concerned that children’s lack of schooling and relationships during quarantine could have a negative effect on their physical and mental health. Evidence suggests that, while at home during quarantine, children engage in less physically activity, suffer from irregular sleeping patterns and follow less healthy diets – all factors that contribute to increased weight and lower cardiorespiratory well-being [8, 9]. Alongside these adverse health effects, psychological stressors may also come into play during extended periods of quarantine. In particular, children may suffer from a lack of personal space, boredom, inadequate information about the quarantine situation and a lack of personal contact with friends, classmates and teachers. The interaction between lifestyle and psychological stressors during quarantine can aggravate negative health effects, in a vicious circle. All of these stressors may have a negative effect on mood and anxiety levels, as well as perceptions of reality.

Within the current context of the COVID-19 pandemic, children, parents and (most frequently) grandparents may all become infected with the virus. Thus, it may be necessary for families to rearrange domestic spaces to ensure isolation and prevent contagion. However, in addition to taking these physical measures, parents must also respond to the fear and suffering of their children, which may add to their own stress. In particular, children may express concern about possible infection, the threat to their family integrity, anxiety about separation from their schoolmates and friends and/or more important concerns about death. Their suffering may be manifested in anger, restlessness, frustration and/or disinterest. It is important that parents monitor and respond to such expressions, as dramatic experiences with high stress (such as the death of a parent) can increase children’s risk of developing a psychiatric disorder later in life [10, 11].

As research has shown, containment measures such as quarantine and isolation in the context of a pandemic or disaster can be traumatizing for both children and parents. Sprang and Silman [12] found that 30% of children and 25% of parents in conditions of quarantine or isolation met the criteria for post-traumatic stress disorder (PTSD), as indicated by the *Diagnostic and Statistical Manual of Mental Disorders, 5th edition* (DSM-5) [13]. Specifically, parents presented with avoidance/numbing, re-experiencing and arousal symptoms. Of the parents who met the PTSD cut-off levels, 85.7% had children who also met the clinical cut-off scores.

During quarantine, it is particularly important that children receive calm, balanced and stable parenting. However, it is not uncommon for parents to experience increased nervousness in these situations, and parents may pass their psychological distress to their children [14]. Parents should strive to maintain affective and firm parenting, facilitate constructive dialogue and encourage responsibility and autonomy in their children through the appropriate use of positive reinforcement. Inappropriate parenting behaviors, such as becoming more protective, granting less autonomy and communicating a sense of imminent danger, may contribute to the development of post-traumatic stress symptoms in children [15]. Thus, parents should try to be realistic, objective and flexible when dealing with their children. Some parents may benefit from additional support in managing their emotional stress, perhaps in the form of education on effective strategies to use with their children (i.e. using suitable language, stimulating emotional and affective expression, maintaining social rules and making behaviors understandable). Under conditions of suffering or emotional distress, cognitive processes cease and no action or behavior can occur. Thus, parents should seek moments of joy, lightness and creativity to share with their children.

As children are constantly exposed to the flow of news about the pandemic, parents should try to talk to them often, explaining the facts and answering any questions their children may have. This may help children manage their fear and anxiety and avoid panic. It may also be helpful for parents to share and encourage individual protective practices, such as physical distance and personal hygiene measures. Finally, the use of age-appropriate formats, including comics and videos, may help children understand the disease.

Despite significant interruptions to daily life – including schooling – during quarantine, parents should seek to maintain a stable and consistent routine with their children. In China, a strong administrative system ensured that emergency homeschooling was rigorously implemented during the COVID-19 outbreak. There, the Ministry of Education estimated that more than 220 million children and adolescents were confined to their homes, including 180 million primary and secondary students and 47 million preschool children [16].

Quarantine can represent a good opportunity to improve parent–child interaction, engage children in family activities and build children’s self-esteem and confidence. With the right parenting approach, family bonds can be strengthened and the psychological needs of children can be respected [17]. Suffering and struggling to recover from a traumatic experience can yield remarkable transformation and positive growth [18]. However, ineffective public health systems and negative economic consequences due to quarantine (e.g. job loss and income reduction) can increase environmental distress and exacerbate some psychological conditions.

A study of psychological resilience after Hurricane Sandy showed that experience of the traumatic event was significantly associated with increased depression and post-traumatic stress; however, it also identified that exposure to community and individual resources worked in tandem to build post-disaster resilience [19]. During quarantine, family social care services and children’s mental health services must work together. Remote (i.e. phone, online) or direct interventions for educational and/or psychological/psychiatric support may be required. In particular, children’s psychological state must be monitored over the long term in order to detect the appearance of any post-traumatic disorder symptoms and intervene quickly to manage them. Furthermore, health districts should distribute simple guidelines on healthy lifestyles, children’s mental health and strategies for living with infection. In this situation the media should play a supporting role. During the talk-show the psychological impact of the restricted daily life habits is underestimated. It is important to support public awareness through the intervention of professionals, child psychologists or psychiatrists, who raise the issue of problems related to the mental health of the most fragile individuals in the population.

## Conclusions

During exceptional and dramatic situations, such as a pandemic, national governments must carefully consider their decision to impose a quarantine in order to stop the spread of the virus. When such measures are enacted, governments must enhance health and social care services in order to make this experience as tolerable and safe as possible.

## Data Availability

Not applicable.

## Abbreviations

DSM-5: Diagnostic and Statistical Manual of Mental Disorders, 5th edition
PTSD: Post-Traumatic Stress Disorder
SARS: Severe Acute Respiratory Syndrome
